# Incidence and Risk Factors of Retinopathy of Prematurity at the Armed Forces Hospital, Southern Region, Saudi Arabia

**DOI:** 10.7759/cureus.92504

**Published:** 2025-09-17

**Authors:** Tamer Shetla, Khalid F Alghadam, Eslam M Abuelsaeed, Ragab S Abdelghany, Mohammed Alomari, Badriah G Alasmari

**Affiliations:** 1 Neonatal Intensive Care Unit, Armed Forces Hospital, Southern Region, Khamis Mushait, SAU; 2 Neonatal Intensive Care Unit, King Abdulaziz University Hospital, Jeddah, SAU; 3 Pediatrics, Armed Forces Hospital, Southern Region, Khamis Mushait, SAU

**Keywords:** incidence, retinopathy of prematurity, risk factors, saudi arabia, screening

## Abstract

Background

Retinopathy of prematurity (ROP) is a vasoproliferative disorder that affects preterm infants, potentially leading to blindness in a considerable percentage of these neonates. This study aimed to determine the incidence of ROP and its associated risk factors at a tertiary eye care center.

Methodology

This study followed a retrospective research design at the Armed Forces Hospital, Southern Region, Saudi Arabia. The electronic hospital records for neonates born from January 2020 to December 2021, with a gestational age <32 weeks, birth weight ≤1,500 g, and at risk of ROP, admitted to the neonatal intensive care unit were investigated, and the incidence of ROP was determined. The associations between risk factors and the development of ROP were assessed.

Results

A total of 166 preterm babies were included (n = 119, 71.7% were males), of whom 33 (19.9%) developed ROP. Significant risk factors associated with ROP included gestational age <28 weeks (p = 0.001), vaginal delivery (p = 0.003), low birth weight (<1,000 g) (p = 0.001), associated intraventricular hemorrhage (p = 0.001), prolonged duration of mechanical ventilation (p = 0.017), and prolonged hospital stay (p = 0.001). A cut-off level of <850 g for newborn’s birth weight had a 71.4% sensitivity and 63.6% specificity, while a cut-off level of <28 weeks for newborn’s gestational age had a 60.9% sensitivity and 75.8% specificity.

Conclusions

Incidence of ROP is high among preterm babies. The main risk factors include gestational age and low birth weight. Therefore, revision of these risk factors is necessary to minimize costs and unduly prolonged hospital stays.

## Introduction

Retinopathy of prematurity (ROP) is a major cause of preventable blindness in preterm infants. It is currently the leading preventable cause of childhood blindness worldwide. The estimated incidence of ROP ranges from 50% to 70% among infants with a birth weight below 1,500 g [1]. Over the past three decades, developed countries have reported a nearly tenfold increase in the incidence of ROP. Between 1990 and 2011, rates of severe ROP increased from 1.7/1,000 to 14.8/1,000 preterm births worldwide [2]. In the United States, it has been estimated that about 1,100 to 1,500 infants develop ROP requiring treatment each year, and 400 to 600 infants will become legally blind due to ROP [3]. The incidence of ROP in Saudi Arabia ranges from 23% to 56% [4]. ROP is characterized by the presence of an avascular retina with consequent aberrant retinal neovascularization. In severe cases, retinal traction and retinal detachment develop, which lead to permanent blindness [1].

Several risk factors have been clarified, such as excessive oxygen supplementation, low gestational age, and low birth weight. The first documented cases of ROP occurred in the late 1940s and were originally termed “retrolental fibroplasia.” These cases were linked to the unregulated use of supplemental oxygen in closed incubators, which, while improving survival rates among preterm infants, also led to a surge in blindness [5]. Although oxygen administration is now more tightly controlled, ROP remains a significant problem, especially among extremely preterm infants (<28 weeks’ gestation) [6]. This persistence is attributed to the immaturity of the retina in these neonates. The absence of essential intrauterine growth factors hampers normal retinal vascular development. Consequently, the timing and levels of postnatal oxygen exposure continue to pose a challenge [6,7]. Several other risk factors have also been identified in the literature. For example, Manzoni et al. reported that neonatal infections, particularly fungal infections, are risk factors for ROP [8]. Tolsma et al. added that late, but not early, neonatal bacteremia is associated with severe ROP in extremely low-gestational-age neonates [9]. The increased risk associated with infection might be partly due to systemic inflammation, which could act synergistically with hyperoxia to mediate the effects of placental infection [10].

Genetic factors might also be a risk factor for ROP. It has been observed that ROP occurs more often in White than in Black infants and in boys than in girls. Genetic polymorphisms might change gene function, which could affect the disease [11]. Many countries have adopted guidelines and screening criteria based on weight and gestational age, and these are modified according to population-based studies on the incidence of ROP [12]. In Saudi Arabia, the practical national guidelines for screening and treatment of ROP recommend screening neonates with a birth weight of ≤1,500 g and/or gestational age of ≤32 weeks [3]. The guidelines of the American Academy of Pediatrics (AAP) recommend that infants with a birth weight of <1,500 g, or a gestational age of <30 weeks or less, should undergo retinal screening examinations using binocular indirect ophthalmoscopy [13]. Good et al. noted that despite the large number of epidemiologic studies aiming to define the possible risk factors for ROP, it is still not possible to adequately predict infants at high risk [14]. Therefore, more specific screening criteria are largely needed as the present screening examinations are stressful both for infants and their caregivers. Moreover, most screened infants do not develop vision-threatening disease [15].

There is a clear need for more refined and specific screening criteria. These would help better identify infants at genuine risk for blinding ROP, while also minimizing unnecessary examinations in low-risk infants. Therefore, this study aims to investigate the incidence and risk factors of ROP among preterm infants admitted to the Armed Forces Hospital, Southern Region, Saudi Arabia. By identifying the prevalence of ROP and analyzing associated neonatal and maternal risk factors, the findings can inform clinical practice, improve early detection, and, ultimately, reduce the burden of preventable childhood blindness in the region.

## Materials and methods

Study design and setting

This retrospective cohort study was conducted in the neonatal intensive care unit (NICU) of the Armed Forces Hospital, Southern Region (AFHSR), Khamis Mushait, Saudi Arabia.

Study population

According to the practical national guidelines for screening and treat­ment of ROP [3], the study population included Saudi infants with a birth weight of <1,500 g, or a gestational age of <30 weeks or less, in addition to infants who displayed an unstable medical course, as determined by the attending neonatologist at the NICU of AFHSR. Neonates were excluded if they were non-Saudi or did not meet our NICU clinical criteria for ROP screening.

Data collection

Data were collected from the hospital electronic records. The hospital records for cases with a birth weight of 1,500 g or less and/or a gestational age of 30 weeks or less who had been screened for ROP according to the national screening criteria were considered. The researcher did not set an a priori sample size, but the present study was rather designed to include data from January 2020 to December 2021, during which neonatology care was practiced.

A structured data collection sheet was developed by the researchers to extract relevant information from the hospital records. Independent variables included infant characteristics such as sex, mode of delivery, birth weight, and Apgar score at five minutes. Maternal variables included gestational age, pre-eclamptic toxemia, and premature rupture of membranes. Associated neonatal morbidities were also recorded, including patent ductus arteriosus, bronchopulmonary dysplasia, and intraventricular hemorrhage. In addition, management-related factors such as antenatal steroid administration, surfactant use, mechanical ventilation, postnatal steroid administration, and hospital stay duration were collected. The primary outcomes of interest were the development of ROP and neonatal mortality. Records with missing data for the primary outcome (ROP status) were excluded.

Statistical analysis

Data entry and analysis were performed using SPSS version 25.0 (IBM Corp., Armonk, NY, USA). Data were analyzed by the chi-square test. Predictions of ROP according to the infant’s birth weight and gestation age were assessed by using the receiver operating characteristic (ROC) curve. Sensitivity, specificity, predictive values, and diagnostic accuracy were calculated. P-values <0.05 were considered statistically significant.

Ethical considerations

This study was approved by the Institutional Review Board of the Armed Forces Hospital, Southern Region (approval number: AFHSRMREC/2022/PEDIATRICS/582). As this study is retrospective, data were collected with a waiver of consent for de-identified data. No authors have a conflict of interest.

## Results

In this study, 71.7% (n = 119) of cases were males. Gestational age of 46.4% (n = 77) was less than 28 weeks, while that of 50% (n = 83) was 28-31 weeks. Almost one-third of cases (32.5%, n = 54) were delivered by cesarean section. Apgar scores at five minutes of 75.3% (n = 125) of cases were >7. The birth weight of 22.3% (n = 37) of cases was less than 750 g, while that of 44.6% (n = 74) was 750-1,000 g, and that of 33.1% (n = 55) was 1,000-1,500 g. Pre-eclamptic toxemia affected 10.2% (n = 17) of our cases’ mothers, while 14.5% (n = 24) had premature rupture of membranes, and 17.5% (n = 29) were small for gestational age. Patent ductus arteriosus was present in 86.7% (n = 144) of cases, bronchopulmonary dysplasia was present in 63.9% (n = 106) of cases, while 18.7% (n = 31) had intraventricular hemorrhage (Table 1).

**Table 1:** Characteristics of the study sample.

Characteristic	N	%
Sex		
Male	119	71.7
Female	47	28.3
Gestational age		
<28 weeks	77	46.4
28–31 weeks	83	50.0
32–33 weeks	6	3.6
Mode of delivery		
Cesarean section	112	67.5
Vaginal	54	32.5
Apgar score at five minutes		
<7	41	24.7
≥7	125	75.3
Birth weight		
<750 g	37	22.3
750–1,000 g	74	44.6
1,000–1,500 g	55	33.1
Maternal pre-eclamptic toxemia	17	10.2
Premature rupture of membranes	24	14.5
Small for gestational age	29	17.5
Patent ductus arteriosus	144	86.7
Bronchopulmonary dysplasia	106	63.9
Intraventricular hemorrhage	31	18.7

**Figure 1:**
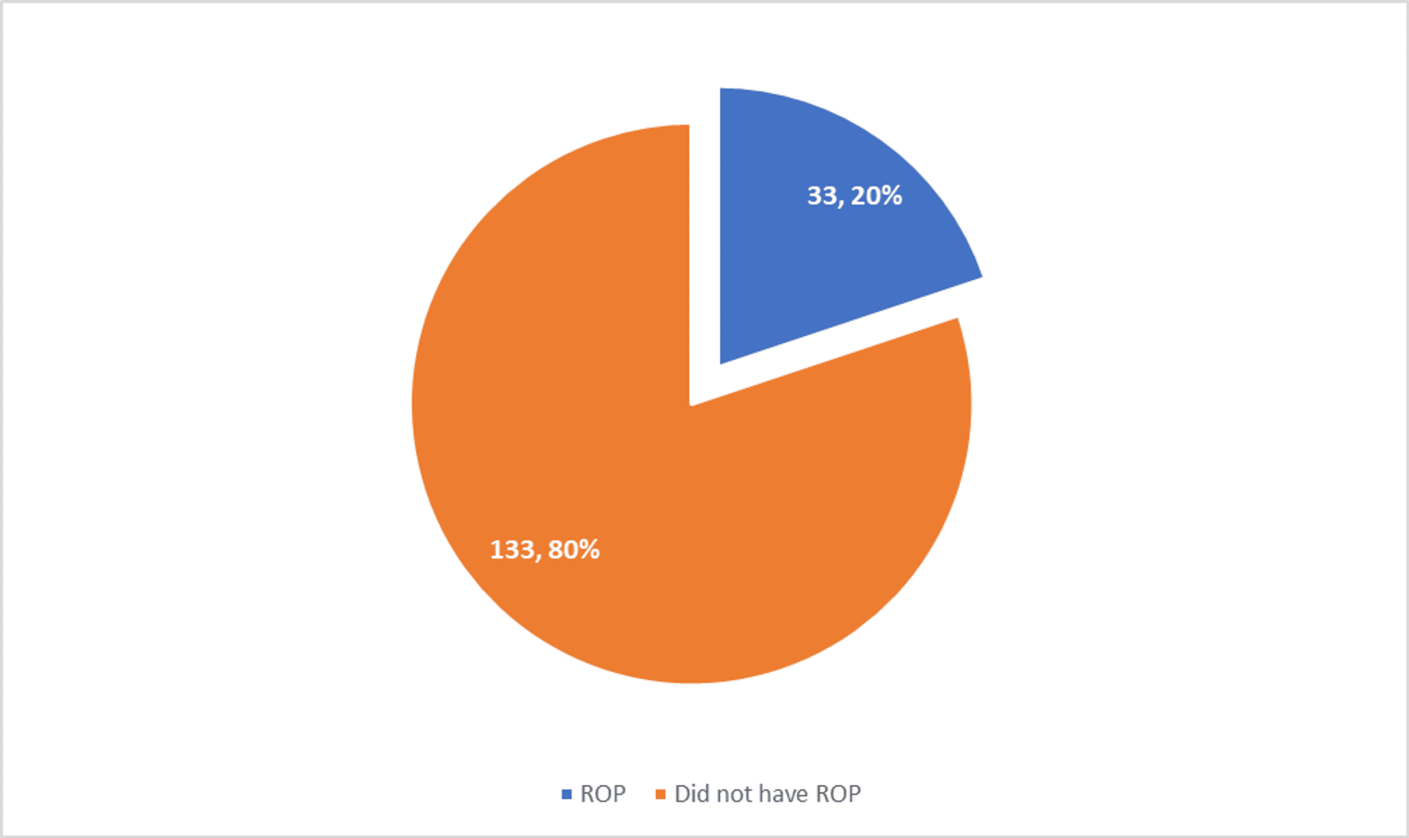
Incidence of retinopathy of prematurity among preterm infants.

Figure 1 shows the incidence of ROP. Overall, 19.9% (n = 33) had ROP, whereas 80.1% (n = 133) did not have ROP.

Antenatal steroids were administered to 64.5% (n = 107) of cases, while 94% (n = 156) received surfactant and 8.4% (n = 14) received postnatal steroids. Duration of mechanical ventilation was fewer than 10 days in 41% (n = 68) of cases, 33.1% (n = 55) received mechanical ventilation for 10-30 days, while it was more than 30 days in 25.9% of cases (n = 43). The hospital stay of 28.3% (n = 47) of cases was less than two months, two to four months for 38.6% (n = 64) of cases, four to six months for 13.3% (n = 22) of cases, and more than six months for 19.9% of cases (n=33). In addition, 29.5% (n = 49) of cases died (Table 2).

**Table 2:** Management and outcome of the study sample.

Characteristic	N	%
Antenatal steroids	107	64.5
Surfactant administration	156	94.0
Postnatal steroids	14	8.4
Duration of mechanical ventilation		
<10 days	68	41.0
10–30 days	55	33.1
>30 days	43	25.9
Duration of hospital stay		
<2 months	47	28.3
2–4 months	64	38.6
>4–6 months	22	13.3
>6 months	33	19.9
Outcome		
Survival	117	70.5
Death	49	29.5

ROP was significantly higher among cases with less than 28 weeks’ gestational age (p = 0.001), among those who were delivered by vaginal section (p = 0.003), and among those whose birth weights were less than 1,000 g (p = 0.001). Moreover, ROP was significantly higher among cases with intraventricular hemorrhage (p = 0.001). However, ROP did not differ significantly according to other characteristics (Table 3).

**Table 3:** Comparison of study groups according to participants’ characteristics. ^†^: Statistically significant. ROP = retinopathy of prematurity

Characteristics	Control (n = 133)		ROP (n = 33)		P-value
	N	%	N	%	
Sex					
Male	97	72.9	22	66.7	0.475
Female	36	27.1	11	33.3	
Gestational age					
<28 weeks	52	39.1	25	75.8	0.001^†^
28–31 weeks	75	56.4	8	24.2	
32–33 weeks	6	4.5	0	0.0	
Mode of delivery					
Cesarean section	97	72.9	15	45.5	0.003^†^
Vaginal	36	27.1	18	54.5	
Apgar score at five minutes					
<7	34	25.6	7	21.2	0.604
>=7	99	74.4	26	78.8	
Birth weight					
<750 g	26	19.5	11	33.3	0.001^†^
750–1,000 g	54	40.6	20	60.6	
>1,000 g	53	39.8	2	6.1	
Small for gestational age					
No	107	80.5	30	90.9	0.157
Yes	26	19.5	3	9.1	
Premature rupture of membranes					
No	111	83.5	31	93.9	0.125
Yes	22	16.5	2	6.1	
Pre-eclamptic toxemia					
No	119	89.5	30	90.9	0.808
Yes	14	10.5	3	9.1	
Patent ductus arteriosus					
No	20	15.0	2	6.1	0.173
Yes	113	85.0	31	93.9	
Bronchopulmonary dysplasia					
No	50	37.6	10	30.3	0.435
Yes	83	62.4	23	69.7	
Intraventricular hemorrhage					
No	115	86.5	20	60.6	0.001^†^
Yes	18	13.5	13	39.4	

Table 4 shows that ROP was significantly higher among cases with prolonged mechanical ventilation for more than 30 days (p = 0.017) and prolonged hospital stays of more than six months (p < 0.001). However, ROP did not differ significantly according to other characteristics.

**Table 4:** Comparison of study groups according to participants’ management and outcome. †: Statistically significant. ROP = retinopathy of prematurity

Characteristics	Control (n = 133)		ROP (n = 33)		P-value
	N	%	N	%	
Antenatal steroids					
No	43	32.3	16	48.5	0.083
Yes	90	67.7	17	51.5	
Surfactant administration					
No	10	7.5	0	0.0	0.104
Yes	123	92.5	33	100.0	
Postnatal steroid					
No	121	91.0	31	93.9	0.584
Yes	12	9.0	2	6.1	
Mechanical ventilation					
<10 days	58	43.6	10	30.3	0.017^†^
10–30 days	47	35.3	8	24.2	
>30 days	28	21.1	15	45.5	
Hospital stay					
<2 months	43	32.3	4	12.1	<0.001^†^
2–4 months	54	40.6	10	30.3	
>4–6 months	20	15.0	2	6.1	
>6 months	16	12.0	17	51.5	
Outcome					
Survival	93	69.9	24	72.7	0.752
Death	40	30.1	9	27.3	

**Figure 2:**
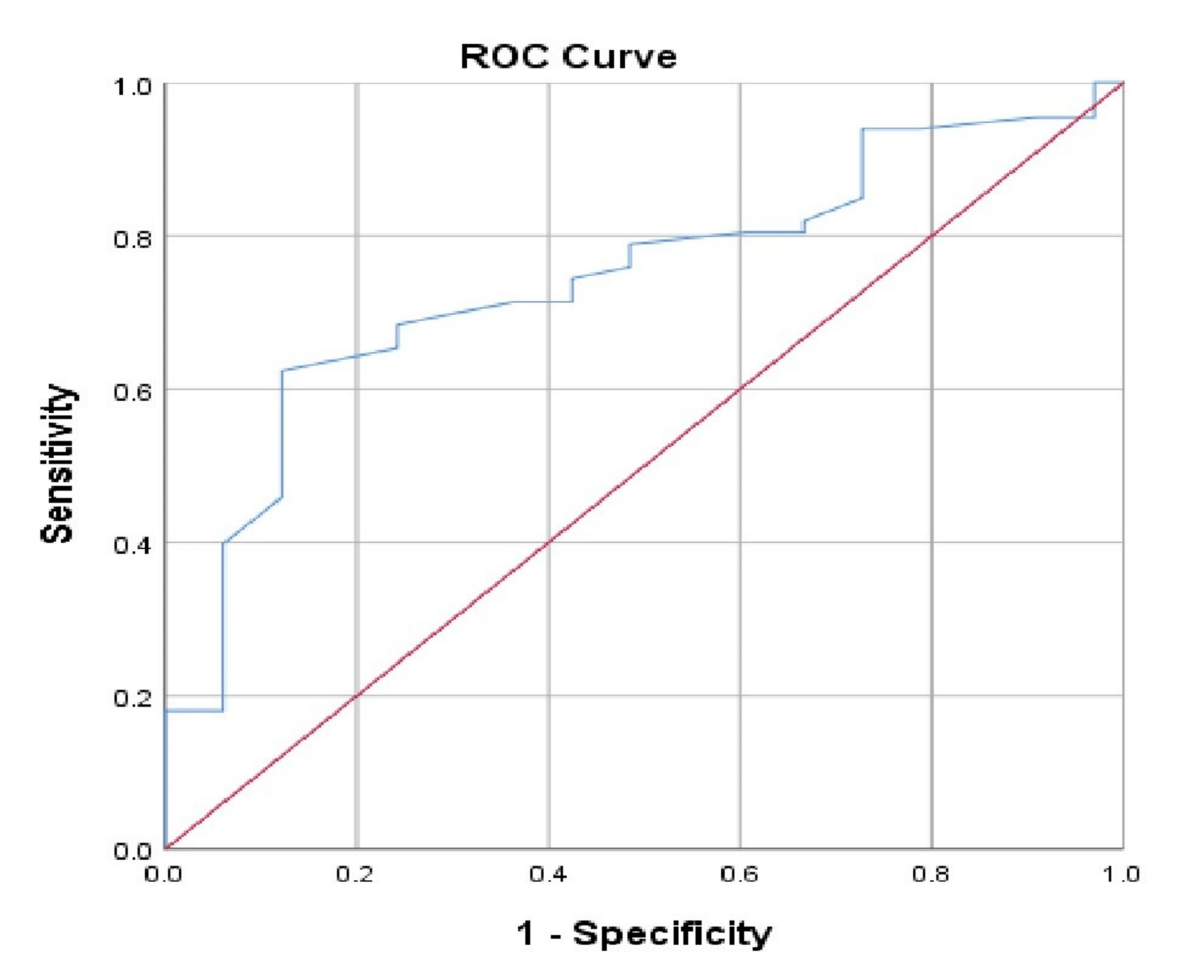
Area under the receiver operating characteristic curve (ROC) for birth weight predicting retinopathy of prematurity.

At a cut-off level of <850 g for newborns’ birth weight, the sensitivity was 71.4% and the specificity was 63.6% (Table 5, Figure 2).

**Table 5:** Validity results for birth weight.

Validity criteria	Result
Cut-off value	<850 g
Sensitivity	71.4%
Specificity	63.6%

**Figure 3:**
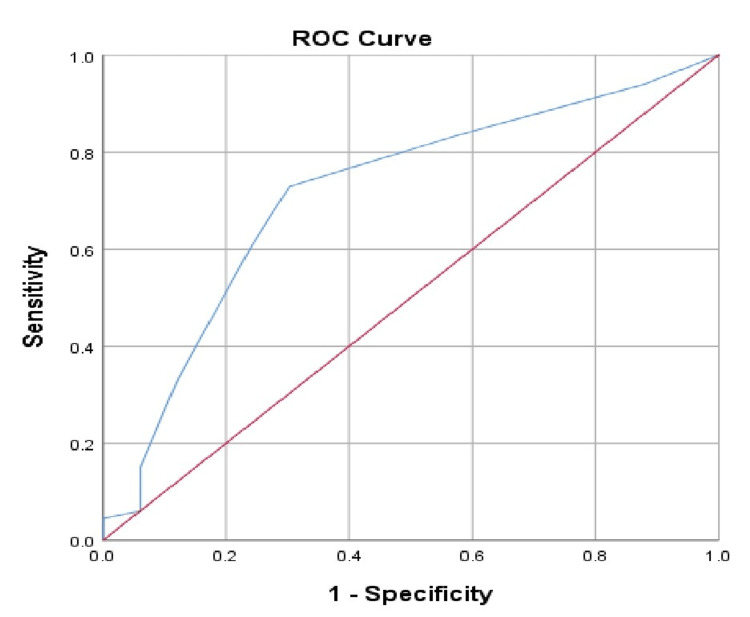
Area under the receiver operating characteristic curve (ROC) curve for gestational age predicting retinopathy of prematurity.

At a cut-off level of <28 weeks for newborns’ gestational age, the sensitivity was 60.9% and the specificity was 75.8% (Table 6, Figure 3).

**Table 6:** Validity results for gestational age.

Validity criteria	Result
Cut-off value	<28 weeks
Sensitivity	60.9%
Specificity	75.8%

## Discussion

The findings of the present study revealed an incidence rate of 19.9% of ROP among study participants. This finding is in accordance with that reported by Yanovitch et al. [15], who stated that about 10% of preterm infants screened for ROP will develop vision-threatening disease. However, it is lower than those reported both locally in Riyadh City by Binkhathlan et al. (56%) [16], regionally, in Oman (34.4%) [17], and internationally, in India (47%) [18]. Of note, our results may reflect a good utilization of the national screening criteria and proper management of preterm infants in our study setting. According to Owen et al. [1], ROP should be screened for, as if undetected, it may result in permanent, life-long blindness. With improved preterm infants’ survival, the incidence of ROP may increase. However, with improved management of preterm infants, its incidence may be lowered. Consistent with that reported in the literature, the present study revealed that early gestational age (i.e., <28 weeks) and low birth weight (i.e., <1,000 g) demonstrated significantly higher incidence for ROP among our cases. The individual significance of early preterm birth compared with low birth weight is supported by other studies. Woo et al. [19] found that in twins with discordant gestational weights, gestational age was a better predictor of ROP.

The present study also showed that significantly more cases of ROP occurred among preterm infants who were delivered vaginally and those who underwent prolonged mechanical ventilation. However, it is important to understand that these findings do not necessarily highlight causality as confounding factors (e.g., perinatal stress, neonatal resuscitation needs) also influence this relationship. Moreover, associated intraventricular hemorrhage was significantly associated with the occurrence of ROP in our cases. However, several other factors in this study were not significantly associated with ROP, such as male gender, Apgar scores, premature rupture of membranes, pre-eclampsia, patent ductus arteriosus, and bronchopulmonary dysplasia. These findings are in accordance with those reported by several studies. Manzoni et al. [20] found that ROP occurred in 40.9% of vaginally delivered neonates and 17.5% of those born via cesarean section (p = 0.008). They concluded that birth by vaginal delivery is a significant predictor of ROP in low-birth-weight infants. Therefore, close ophthalmological surveillance should be provided for preterm low-birth-weight infants born vaginally. Other epidemiologic studies have suggested additional risk factors for ROP, including mechanical ventilation, maternal pre-eclamptic toxemia, intraventricular hemorrhage, presence of patent ductus arteriosus, and male gender [1,21-23].

Despite the provision of proper management for our preterm infants, the case fatality rate was quite high (29.5%). This outcome is expected as preterm births are the most common cause of neonatal mortality and constitute the second most common cause of death in children younger than five years [24]. A nationwide study from Korea reported that the mortality rate was 4.8 per 1,000 person-years in ROP patients included in their study [25]. Similarly, in another study from Germany, the mortality rate for infants with ROP was 60.33 per 10,000 [26]. The utilization of the national screening criteria by the present study showed variable validity measures. At a cut-off level for newborns’ birth weight at <850 g, sensitivity was 71.4% and specificity was 63.6%. On the other hand, a cut-off level for newborns’ gestational age at <28 weeks resulted in a higher specificity (75.8%) but lower sensitivity (60.9%). Ying et al. [22] noted that the current screening guidelines provide relatively high sensitivity but with low specificity. Owen et al. [1] stressed that work to model risk factors and improve ROP prediction has been complicated by its multifactorial nature. Current statistical modeling for ROP risk has shown variable sensitivities across different populations.

Strengths and limitations

The main strength of this study is a comprehensive analysis of most ROP risk factors. Moreover, the current study cohort received uniform clinical care over a two-year time period, which strengthens the ability to identify individually significant ROP risk factors. However, some limitations should be considered while interpreting the findings. First, the current study followed a retrospective research design. Therefore, it can only provide estimates for the incidence of ROP in our tertiary care study setting. Moreover, in an effort to include only infants with uniform neonatal care, the study was not conducted in multiple settings, and our sample included only Saudi infants. Therefore, our sample size was limited. However, the assessment of a homogeneous population is expected to reduce the likelihood of spurious associations. Furthermore, we did not perform multivariate analysis, which limits the ability to find cofounding factors in the study.

## Conclusions

Based on the findings of the present study, it can be concluded that the incidence of ROP is high among preterm babies. The identification of risk factors for ROP development is crucial for improved management and blindness prevention among infants. Our findings confirm the accepted significance of early birth and low birth weight as risk factors for ROP. Vaginal delivery and associated intraventricular hemorrhage are additional risk factors for ROP. A cut-off level of <850 g for newborns’ birth weight provided 71.4% sensitivity and 63.6% specificity, while a cut-off level of <28 weeks for newborns’ gestational age provided 60.9% sensitivity and 75.8% specificity. Our results reflect the benefits of following the national screening criteria and proper management of preterm infants in our study setting. It is recommended to follow the national screening criteria and properly manage preterm infants. However, revision of risk factors is necessary to minimize costs and unduly prolonged hospital stays. Future work within a larger, more diverse population is necessary for a better understanding of the validity of the identified risk factors for ROP.
